# Data distribution in public veterinary service: health and safety challenges push for context-aware systems

**DOI:** 10.1186/s12917-017-1320-0

**Published:** 2017-12-22

**Authors:** Laura Contalbrigo, Stefano Borgo, Giandomenico Pozza, Stefano Marangon

**Affiliations:** 1 Istituto Zooprofilattico Sperimentale delle Venezie, Viale Dell’Università 10, 35020 Legnaro, (PD) Italy; 2 Laboratory for Applied Ontology, ISTC-CNR, Via alla Cascata, 56/C, 38123 Trento, Italy

**Keywords:** Public veterinary services, Institutional veterinary context, Data user, Data distribution, Ontology

## Abstract

**Background:**

Today’s globalised and interconnected world is characterized by intertwined and quickly evolving relationships between animals, humans and their environment and by an escalating number of accessible data for public health. The public veterinary services must exploit new modeling and decision strategies to face these changes. The organization and control of data flows have become crucial to effectively evaluate the evolution and safety concerns of a given situation in the territory. This paper discusses what is needed to develop modern strategies to optimize data distribution to the stakeholders.

**Main text:**

If traditionally the system manager and knowledge engineer have been concerned with the increase of speed of data flow and the improvement of data quality, nowadays they need to worry about data overflow as well. To avoid this risk an information system should be capable of selecting the data which need to be shown to the human operator. In this perspective, two aspects need to be distinguished: data classification vs data distribution. Data classification is the problem of organizing data depending on what they refer to and on the way they are obtained; data distribution is the problem of selecting which data is accessible to which stakeholder. Data classification can be established and implemented via ontological analysis and formal logic but we claim that a context-based selection of data should be integrated in the data distribution application. Data distribution should provide these new features: (a) the organization of situation types distinguishing at least ordinary vs extraordinary scenarios (*contextualization of scenarios*); (b) the possibility to focus on the data that are really important in a given scenario (*data contextualization by scenarios*); and (c) the classification of which data is relevant to which stakeholder (*data contextualization by users*).

**Short conclusion:**

Public veterinary services, to efficaciously and efficiently manage the information needed for today’s health and safety challenges, should contextualize and filter the continuous and growing flow of data by setting suitable frameworks to classify data, users’ roles and possible situations.

## Background

The increasing interdependence between humans, animals and their products as well as the close association with companion animals have encouraged a change in the public health system thinking [1]. The study of public veterinary systems has rapidly grown as a domain in itself supported by the adoption since 1984 of the “One Health” paradigm as an effective strategy for the prevention and control of zoonoses [2, 3]. In an increasingly globalised world, this new approach encompasses zoonotic infections, food safety, the environment and the health delivery systems. The integration of the epidemiological and economic frameworks within the new technological turn called the *Internet of Things* (IoT), led to an escalating number of distributed sensors, which, together with the laboratory data, make available large amounts of information [4]. Indeed, health management systems are fully part of the so-called *big data environment* [5]. Nowadays international organizations like FAO and OIE are developing web based applications to collect, store and analyze data from different sources with the aim to combine epidemiological, spatial and genetic data as well as data about farming systems in order to provide appropriate information to public authorities and scientists on the emergence and spread of animal diseases [6]. This huge amount of data requires a methodological approach to effectively assess and manage the health and safety of the ecosystems [7].

This is a significant challenge, which calls for efficient public veterinary data management systems, for quick and focused data distribution as to better help the stakeholders in their understanding of the situation and decision-making processes [8–10].

Every day, public veterinary managers, as well as other stakeholders like farmers, food producers, veterinarians and distributors deal with huge quantities of data on a variety of topics and different organization quality, often with several types and forms. These actors need to use them to understand scenarios in the territory and to make decisions to manage both ordinary and emergency situations. Here, we use the term ‘situation’ to indicate the state of an area or environment of interest as it is at some point in time. Situations can be classified as positive, negative or neutral by each stakeholder, depending on its role and interests, distinguishing, in particular, between ordinary ones (positive or neutral) and emergency ones (negative).

In ordinary situations, surveillance, monitoring and eradication plans are the basis of veterinary public health. The implementation of these entails the need to elaborate the data collected during previous programs and to integrate them with the new epidemiological information. This is crucial to make an effective risk assessment and to decide on activity plans finalized to guarantee the achievement of sanitary goals with minimum cost and risk for the consumers, the producers and the public veterinary system itself [11–13].

Analogously, the management of emergency situations (*mitigation and prevention -* e.g., risk mapping and hazard identification; *preparedness and planning* - e.g., contingency planning, personnel training, warning system; *response and recovery* - e.g., contingency plan activation, epidemiologic surveillance) [14] requires to look at different information types integrating several data sources to correctly identify the kind of incident, the location, the magnitude and to monitor the evolution of the situation (e.g., geographical coordinates, logistic and environmental data, animal, means of transport and people movements, laboratory data). For instance, in veterinary epidemic emergencies the identification of outbreaks and their eradication can be managed effectively only when the competent authorities have quick access to reliable, well processed and pre-analysed information on health-related data [15, 16]. This information should be coherently and rapidly organized and presented in a very intuitive way to be quickly assimilated and analysed by public health managers for decision-making. Otherwise, doubts about the real situation can arise with delay in critical decisions. In the worst-case scenario, data can be distributed in a scattered way, leading to missing information and even misinterpretation [17]. The successful management of a veterinary control system depends on the quality of the data as well as on their correct interpretation. The organization of the data distribution with respect to the targeted data user is clearly a key factor [18–20].

Therefore, to be efficient, a veterinary data management system should comply with the needs of the different stakeholders that operate in the same territory although with different perspectives: monitoring, surveillance, screening and modeling of infectious diseases, prevalence estimation, risk-factor study, quantitative risk assessment, product evaluation, commercialization and so on. Each stakeholder is interested in the goals related to his/her duties and these can be quite apart going from health hazard and commercialization approval to animal health monitoring. The stakeholder’s purposes indicate his/her interest in the situation and the subset of data relevant to him/her. Furthermore, when two stakeholders need the very same data, they might require them in different formats or granularity. Moreover, the very same data can acquire different connotations: they can be considered positive by one actor and negative by another [21].

Finally, the meaning of data is often determined in comparison with other data [22]. For example, during an Avian Influenza outbreak in a poultry farm, the poultry producer focuses on the pass/fail result of the laboratory test because this may lead to the stamping out of the entire flock. Instead, the public authority evaluates the same data against a larger period of time, a broader spatial region, considering virus genetic features, comparing this information with data originating from other farms or connected production sites and considering a variety of possible epidemiological scenarios [23–25].

These observations show that data interpretation can be challenging and that the traditional approach based on comprehensive reports collecting large numbers of heterogeneous data cannot be a solution anymore, especially if the data relevant for the user are spread across the whole document and reported using granularities not promptly comparable [26, 27]. Of course, beside data interpretation there are other elements that influence the manager in the decision-making process, e.g., scientific background, regulations, time constraints, experience on the field, economic as well as socio-political considerations [28, 29]. While recognizing the importance of the other elements within the whole veterinary system, we claim that there is the need to improve the impact and to correct the interpretation of data taking into account the context in which they are collected and the role of the actors that read them [30]. For these reasons, it is time for the public veterinary data management system to exploit new data modeling and data distribution strategies that can match today’s information needs.

Data misleading due to wrong contextualization, distribution of information or representation is a daily problem within the big data environment in public health [31, 32]. Many examples are reported in both human and veterinary medicine. Especially computational epidemiology needs large and flexible datasets for epidemiological models based on records of variable forms and origins (e.g., types of individuals at specific locations and points in time, records of movement of individuals between locations, social media data, digital traces and results of diagnostic test and genomic analysis) [33]. For example, EMPRES-i (FAO’s global animal disease information system) offers mapping and graphic functions combining data coming from different databases that do not use the same references to describe host taxonomy and geographical locations, therefore, at the beginning, it was not possible to establish an accurate correspondence between the related references [34]. Moreover, in the veterinary domain, *geographical information systems* (GIS) required spatial data of animal holdings. Often these records lack relevant information, leading to data incompatibility and even inconsistency [35]. Furthermore, various techniques need to be used to enhance decision-making process in the face of data uncertainty as happens when developing information systems based on sparse and incomplete datasets of varying quality as for example the case of GLiPHA (Global Livestock Production and Health Atlas) [36]. Likewise in human medicine, epidemiologists face similar concerns when mapping raw data in epidemiological studies like cancer mortality rate: the generation of spurious spatial features and statistical artifacts may lead to spurious spatial pattern [37, 38]. Regarding misleading interpretation of statistical analysis results, in oncologic clinical trials, it is necessary to identify accurate outcomes and endpoints as well as censoring in the study, otherwise statistical analyses can easily fail [39]. In biology the rapid increase in data volumes due to new molecular technologies like the *next-generation sequencing* (NGS), and the rise of omics data generate new data types and it is still unclear how to process, store and integrate them to produce usable knowledge in accessible formats for healthcare professionals [40–42].

It is evident that the selection of the data to distribute, the granularity of the information and the outline of the report should start from the goals of the targeted stakeholder, including its needs and education [43]. Moreover, following the trend of the IoT [44] and the large use of *Information Technology* (IT) devices like “*precision livestock farming*” (PLF), we cannot ignore the problem of integrating and understanding data generated for specific purposes using different sensors and methodologies by farmers, producers and dealers (e.g., cow pedometer, environment sensors, data-logger, video-taping/recording, sensors installed in moving devices, accelerometer) [45–48]. In this framework it is essential to merge these data using reliable methodologies based on typology and provenance as well as device capacities, location and trustability. While typology and provenance are successfully analyzed via the application of ontological frameworks, see e.g. [49–51], contextualization techniques for the distinct stakeholders need to be better understood. As an example, Livestock Geo-wiki, a platform that aims to develop a global livestock information system, provides innovative visualization and analysis tools addressing specific requirements of different groups of users but it does not consider contextualization techniques focused on users’ need classification [52].

By introducing a systematic approach for data classification and for the delineation of the stakeholders’ perspective, we can deepen the accuracy of data distributed to the stakeholders, like sensitivity and specificity [53], increase their data understanding and trust, and at the same time reduce the risk of data overflow.

The outcomes of the study and contextualization of data have also a positive impact on the integration of information from various datasets, which can be harvested for new purposes beyond early detection of animal diseases or food safety issues, like epidemiological studies, pharmacovigilance or identification of emerging risks [54, 55]. Finally, data contextualization increases awareness of the potential risks and ethical challenges related to data sensitivity, legitimacy requirements, and the relationship between ethics and methodology [56].

## Main text

### The need of data selection strategies in the public veterinary management system

The public veterinary data management system is an important actor in the territorial network of stakeholders since it functions as an information hub from which the different users receive updated data. This flow of information from the public veterinary data management system and the stakeholders ensures that each actor has a proper understanding of the situation in the field. The core activities of this management system, for what concerns us here, are the acquisition, integration, elaboration, classification and distribution of veterinary data in the field of food safety, animal health and welfare. Information systems are essential to run public veterinary services since veterinary monitoring and surveillance are largely data-oriented to the point that the term *big data environment* has become paradigmatic even in this domain [57, 58]. Public veterinary data management systems have been working with a traditional approach: they collect information from different sources (like sample collectors, public officers, or their own laboratories) and release it to the stakeholders via the use of reports. Reports are data sets organized according to formats fixed once for all. Although there are a few exceptions, mainly due to increased sensibility to privacy concerns, the principle is pretty clear: any data request type is associated with a report type. The same report is released to all the stakeholders no matter the context, their roles or the situation in the field. In short, the management system has no discretional option: it must distribute to every interested party all data relative to a request using some pre-established format.

Today the IT evolution and the new challenges due to the IoT and the so-called big data issue, require to re-assess the public veterinary system organization and strategies. With the exponential grow of data, it is easy to be overwhelmed by the distributed amount of information that has the inherent risk of overpassing the cognitive capacities to correctly read, understand, compare and evaluate them [59]. One could hope that the increasing number of data remains a problem at the IT level since ad hoc services for data filtering (e.g., customization at the client’s side or online search facilities) could help to select the needed information for each evaluation and decision step. If that were true, each stakeholder would still want to access all the available information on a given scenario and use its own services to select what to focus on. Unfortunately, this view is naïve. Data are complex entities that have different meanings when looked at in isolation or in collections. Furthermore, the way data are organized, impacts the narrative they give of the territory. For example OpenFluDB, a large database of influenza virus sequences, is isolate-centric rather than sequence-centric like other influenza virus resources. This choice makes easier the association with a comprehensive amount of clinical and epidemiological data [60].

If we rely on services at the stakeholders’ side, we cannot guarantee a coherent reading of the situation across the stakeholders in the territory. The risk is that incompatible interpretations may be adopted leading to implement mutually contradictory action plans. The organization of a coordination level across stakeholders could solve this last issue, but it is not practical for at least two reasons. 1) stakeholders (e.g. producers and public officers) have different goals and each would try to enforce its own reading of the data; 2) a coordination level adds an extra lapse of time which is a luxury we cannot afford in emergency situations.

As said, reports with large amounts of data are difficult to read since the user has:

- to look at different sections;
- to identify the subsets of data relevant to its role and knowledge;
- to rewrite them in the granularity of interest;
- to compare these data;
- to find a narrative that explains the data coherently with the territory type and history.

This is especially hard when the user is under pressure, like in emergency scenarios and there is no time to translate from the different standards for data management or even to make confident comparisons and integration of data coming from different data sets with changing measure units, density, frequency, etc. More subtlety, the information in the reports is presented from the general perspective of the veterinary service and considers neither the role and goals of the stakeholders nor the context and motivations for which data have been collected. For example, data on samples collected in a monitoring program for dairy farms in a certain area cannot be reliably used to define the health status of the bovine population in the same area including beef livestock. The correct integration of these two types of data requires knowledge that the stakeholder may not have. To reduce the risk of misjudgment and of missed information, we need to investigate which data should be presented to a given user, in which order and in which format. To answer this, one has to develop methodologies for data selection. Data selection can be done in different ways, e.g., task or function oriented. A selection is task-oriented when only data relevant for a determined task (e.g. decision making) are presented to the operator. The selection is functional when the information is shown depending on the general role the operator has in the system (e.g. surveillance). However, data selection is not an isolated step. Two further steps need to be considered: data classification and data distribution. Data classification is the problem of organizing data according to what they refer to and to the way they are obtained. Data distribution is the problem of choosing which data to distribute to which stakeholder and in which context. Data distribution can be seen as the specific use of a data selection methodology.

### Focus on stakeholders’ classification

In the context of the public veterinary data management system, the classification of data and metadata, the data selection methodologies and the data distribution should be guided by the stakeholder’s technical knowledge, role and goals. Note that the role of the user constrains its goals, yet the goals must be explicitly included in the data management since they can change depending on contextual information like available resources. Technical background, role and goal are thus the factors that can be used to balance the tension between full vs limited disclosure in order to optimize data understanding and reduce data overflow. This leads to the issue of user’s classification. If the classification of data is based on what data are about and how they are collected, the classification of users is based on their role in the system. These classifications are not affected by the context and are independent from the particular scenario. This means that one can apply the methodologies developed in the applied ontology area to provide guidelines and criteria [49–51].

Regarding data selection and distribution, we now look at the management of veterinary laboratory data. Laboratory data furnish a fairly simple case without exogenous factors that could hinder the understanding of the problem. To justify and ground the selection and distribution of data, let us assume that a suitable framework for the classification of the stakeholders is available. This framework should be based on the profiling of the user’s types, use this profiling to collect the user’s requirements and to characterize their perspectives as well as aims [61, 62]. Given this, the veterinary management system can prepare a data selection methodology for laboratory data that, depending on the interests, action capacities, technical knowledge and goals of the stakeholder, reasons on the usefulness and completeness of some data set for that user. Note that the goal is not to hide information to the user. On the contrary, the goal is to organize and highlight the data relevant to that user so to put him/her in the best position for data comparison and understanding as it happens using the support of data warehouse in the decision making process [63–65].

The laboratory data example shows also one point we introduced earlier: the need for context classification. Several research communities have been discussing the use of contexts [66, 67] and yet no general approach has been developed since proposals like [68–70] are limited to some application domain or to some types of scenario only. In the case of veterinary services, the context should tell the characteristics of the stakeholders in terms of rights to know, official goals and duties, possibly even information on the procedures it applies and actions it can take. This information should be integrated with the situation in which the stakeholder operates, that is, its environment.

### The notion of context and its impact on the assessment of data knowledge

Being the points cited in the previous paragraph the core elements that a notion of context should manage, a recent proposal by Mizoguchi et al. [71], developed in the area of function context, could help. Mizoguchi and collaborators aim to introduce a general notion of function that applies to both the natural and the engineering domains. They distinguish between three kinds of contexts: systemic, use and design context. The systemic context looks at functionalities of a component from the viewpoint of the system where it is embedded; the use context looks at functionalities of a device in the scenario where the device is used; the design context looks at functionalities of a device from the perspective of the device’s designer. The notion of context in the framework of veterinary public health systems can build upon Mizoguchi’s notion of use context. The use context is associated with a situation and fixed by an intentional agent, which is a participant in the situation aiming to reach some goal(s). The context is understood as an event where a device, a goal, an agent and the environment are at least partially known. The use context is thus a complex entity analyzed from the user’s perspective to identify what function a device (a natural or artifactual part) performs in a certain environment [72].

A veterinary situation presents important differences with respect to Mizoguchi assumptions. Veterinary public health systems deal with information in reports, not physical objects. Furthermore, the process from data collection (like diagnostic activities) to data distribution (like a report release) is not aimed to change the physical world: it starts with the completion of a laboratory test and ends with the users’ knowledge update. In this view, we can think of laboratory data and metadata like *information devices* in veterinary public health systems that are used to change not the physical reality but the users’ knowledge of physical reality. The analogy is between acting on the world to achieve a physical change and acting on the mind to achieve a knowledge change. This match between user contexts in functional studies and user contexts in veterinary health systems suggests that the data provided by the laboratory (the epidemiologic unit, etc.) have a precise functional role: they make possible to realize the needed changes in the user’s mind in terms of situation’s knowledge and awareness. The distribution of a set of data, with a suitable format and organization for a user, impacts the user’s knowledge about the actual situation and, subsequently, on how the user can act to fulfill its role and duties. In other words, a veterinary management system looks as an agent whose goal is to provide data users with an understanding of the actual situation suitable and reliable for their goals and perspectives, and whose tools are data and metadata which must be carefully tuned (that is, selected and distributed) to achieve the desired result. Following the terminology in [71], we call this special user context the *institutional veterinary context.*

Assuming a data user classification and characterization system is available, covering institutional user(s) and consumers, producers and all legal and economic stakeholders, for each agent the data provider has to select the data and metadata to distribute, to decide the granularity of the data, and to organize the report format. This choice of data selection and distribution is driven by the classification of the data and of the users and by an evaluation of the data and metadata that they expect for their interests and activities including their optimal organization [73–75].

Given this setting, data set can be qualified as follows. A data set is *knowledge complete* for a user type in a situation if from this data set and from standard background knowledge for that user type, one can derive how much the area is departing from its standard and acceptable states, the possible risks as well as short and long term consequences. A data set is *action complete* for a user type in a situation if from this data set and from standard background knowledge for that user type, one has all the knowledge needed to develop an optimal plan of actions to achieve its goals [76, 77]. Note that knowledge and action completeness are distinct: the first focuses on understanding the situation and how it may evolve, the latter takes into consideration the user’s aims and its action capacities.

Furthermore, we can say that a data set is knowledge (or action) *minimal* for a data user if none of its proper subsets is knowledge (action, respectively) complete for that user in the sense defined above. Then, institutional veterinary contexts help to assess data knowledge and action completeness and minimality for a user by setting a framework to reliably verify effects of data awareness on the user’s decisions/actions in different scenarios.

**Fig. 1:**
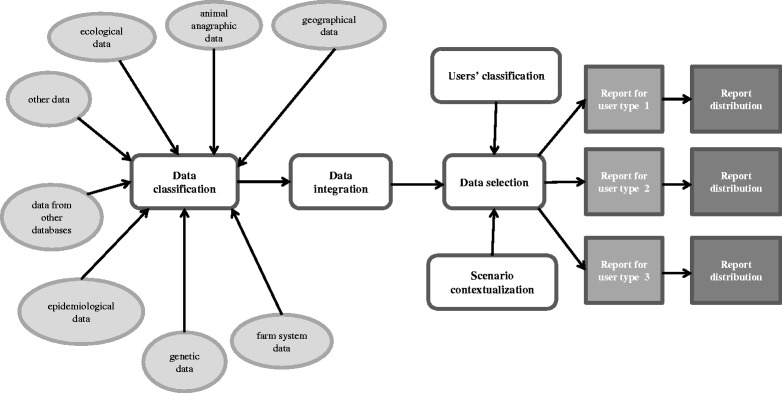
Example of a data flow diagram with a flexible data distribution in accordance with users’ classification and scenario contextualization

Let us call *controlled* a situation in which the data set provided by the veterinary data management system is knowledge and action complete. Recall that this means that the user can understand the situation in the area of interest and has all the information to act optimally for its goals. Note that a controlled situation is just a situation for which we know how to optimize data flow. The situation itself can be ordinary (positive or neutral) or an emergence (negative). Situations that are *uncontrolled* are the most dangerous: they are either wrongly classified or wrongly estimated to the point that the stakeholder makes wrong decisions and the consumers, animals and environment could be exposed to excessive risks, erratic behaviors of the market operators, and unnecessary economic losses. A flexible data integration and distribution, i.e., the selection of data to distribute contextualized to user types, data interrelation and previous knowledge of the situation, can drastically reduce the class of uncontrolled situations [78, 79]. The flexible integration of data and their contextualization are new conceptual tools that can turn *uncontrolled* situations into *controlled* ones while increasing awareness and confidence in the data users. We call *contextualized* the data of a report filtered to match the interests of a specific user, presented depending on the situation at stake and provided with the needed granularity and organization (Fig. 1). When data are contextualized, the data received by a user are even less prone to be misinterpreted, they can effectively help to properly evaluate the risks and to take the correct decisions.

In conclusion, modern veterinary data management systems should include services like data classification, selection and distribution that include these further contextualization features: (a) the organization of situation types starting from the distinction between ordinary vs emergence scenarios (*contextualization of scenarios*); (b) the possibility to focus on the data that are important in a given scenario (*data contextualization by scenarios*); and (c) the classification of which data is relevant to which stakeholder (*data contextualization by users*). With this approach, veterinary data providers can become active information hubs enabling the generation of sustainable and optimized data flows suitable for today’s information age based on IoT and big data. While the advantages of this approach are clear, further discussions are needed to analyze how the veterinary management system would change in relation to, e.g., ethical challenges like privacy concerns and the balance between public health and the individual rights [80, 81].

## Short conclusion

In the last decades, the study of health management systems has rapidly increased embracing the public veterinary services. We claimed that these services, to efficaciously and efficiently manage the information needed today, must include classification and contextualization techniques to filter the increasing and continuous flow of data. Such a step can improve the overall quality of the decision processes since these are highly sensitive to the quantity, quality and organization of the received information. This new approach may become particularly important in emergency situations since today the stakeholders need to quickly realize and assess the risk situation while being engaged in assessing large amounts of incoming data. Therefore a coherent and principled notion of context is essential to help filter and promptly distribute the right information to each user. We introduced the notion of institutional veterinary context, among others, which sets a framework to assess the different techniques for flexible data classification, selection and distribution taking into account contextual perspectives.

## Abbreviation

GIS: Geopraphical Information System
IoT: Internet of Things
IT: Information Technology
NGS: Next-Generation Sequencing
PLF: Precision Livestock Farming

## References

[1] Van Olmen J, Marchal B, Van Damme W, Kegels G, Hill PS (2012). Health systems frameworks in their political context: framing divergent agendas. BMC Public Health.

[2] Bidaisee S, Macpherson NLC. Zoonoses and one health: a review of the literature. J Parasitol Res. 2014; 10.1155/2014/874345. 10.1155/2014/874345 PMC3928857 24634782

[3] Dahal R, Kahn L. Zoonotic diseases and one health approach. Epidemiology. 2014; 10.4172/2161-1165.1000e115.

[4] Simonsen L, Gog JL, Olson D, Viboud C. Infectious disease surveillance in the big data era: towards faster and locally relevant systems. JID 2016; doi:10.1093/infdis/jiw376. 10.1093/infdis/jiw376 PMC5144901 28830112

[5] Lee EC, Aher JM, Goldlust S, Kraemer JD, Lawson AB, Bansal S. Mind the scales: harnessing spatial big data for infectious disease surveillance and inference. JID. 2016; doi:10.1093/infdis/jiw344. 10.1093/infdis/jiw344 PMC5144899 28830109

[6] Farnsworth ML, Hamilton-West C, Fitchett S, Newman SH, de La Rocque S, De Simone L (2010). Comparing national and global data collection systems for reporting. Outbreaks of H5N1 HPAI. Prev Vet Med..

[7] Cocker R, Rushton J, Mounier-Jack S, Karimuriba E, Lutumba P, Kambarage D (2011). Towards a conceptual framework to support one-health research for policy on emerging zoonoses. Lancet Infect Dis.

[8] Marabelli R (2003). The role of official veterinary service in dealing with new social challenges: animal health and protection, food safety, and the environment. Rev Sci Tech OIE.

[9] Capua I, Alexander DJ (2006). The challenge of avian influenza to the veterinary community. Avian Pathol.

[10] Wendt A, Kreienbrock L, Campe A (2015). Zoonotic disease surveillance – inventory of systems integrating human and animal disease information. Zoonoses Public Health.

[11] Jameson LJ, Medlock JM (2011). Tick surveillance in great Britain. Vector Borne Zoonotic Dis.

[12] Vrbova L, Patrick DM, Stephen C, Robertson C, Koehoorn M, Parmley EJ, Galanis E (2016). Utility of algorithms for the analysis of integrated salmonella surveillance data. Epidemiol Infect.

[13] Umhang G, Comte S, Hormaz V, Boucher JM, Raton V, Favier S, Boué F. Retrospective analyses of fox feces by real-time PCR to identify new endemic areas of Echinococcus multilocularis in France. Parasitol Res. 2016:1–5. 10.1007/s00436-016-5220-1 27517858

[14] Cova TJ, Longley PA, Goodchild MF, Maguire DJ, Rhind DW (1999). GIS in emergency management. Geographical information systems: principles, techniques, applications and management.

[15] Iannitti S, Savini L, Palma D, Calistri P, Natale F, Di Lorenzo A (2014). An integrated web system to support veterinary activities in Italy for the management of information in epidemic emergencies. Prev Vet Med..

[16] Drewe AJ, Hoinville LJ, Cook AJC, Floyd T, Gunn G, Stärk KDCSERVAL (2015). A new framework for the evaluation of animal health surveillance. Transbound Emerg Dis.

[17] Dusse F, Jùnior PS, Alves AT, Novais R, Vieira V, Mendoça M (2016). Information visualization for emergency management: a systematic mapping study. Expert Syst Appl.

[18] Dorea CF, Sanchez J, Revie CW (2011). Veterinary syndromic surveillance: current initiatives and potential for development. Prev Vet Med.

[19] Dupuy C, Bronner A, Watson E, Wuyckhuise-Sjouke L, Reist M, Fouillet A (2013). Inventory of veterinary syndromic surveillance initiatives in Europe (triple-S project): current situation and perspectives. Prev Vet Med..

[20] Daniels P, Poermadjaja B, Morrissy C, Ngo TL, Selleck P, Kalpravidh W (2014). Development of veterinary laboratory networks for avian influenza and other emerging infectious disease control: the southeast Asian experience. EcoHealth.

[21] Fürber C, Fürber C (2016). Data Quality. Data quality management with semantic technologies. Wiesbaden: springer Fachmedien.

[22] Clarke R (2016). Big data, big risks. Inf Syst J.

[23] Martell-Moran NK, Mauer WA, Kaneene JB (2011). Assessment of avian influenza surveillance and reporting needs of stakeholders in Michigan, 2007. J Am Vet Med Assoc.

[24] Comin A, Klinkenberg D, Marangon S, Toffan A, Stegeman A (2011). Transmission dynamics of low pathogenicity avian influenza infections in Turkey flocks. PLoS One.

[25] Dalla Pozza M, Ceolin C, Marangon S (2008). Emergency response following suspicion of an avian influenza outbreak. Zoonoses Public Health.

[26] Ferraro S, Braga F, Panteghini M (2016). Laboratory medicine in the new healthcare environment. Clin Chem Lab Med.

[27] Huang L, Fernandes H, Zia H, Tavassoli P, Rennert H, Pisapia D, Elemento O. The Precision Medicine Knowledge Base: an online application for collaborative editing, maintenance and sharing of structured clinical-grade cancer mutations interpretations. BioRxiv. 2016:e059824. doi:10.1101/059824.

[28] Giangaspero M, Sekiguchi S (2016). Risk assessment of animal infectious diseases and decision making process. Clin Microbiol.

[29] Del Giudice M, Caputo F, Evangelista F. How are decision systems changing? The contribution of social media to the management of decisional liquefaction. J Decis Syst. 2016:1–13.

[30] Dey AK (2001). Understanding and using context. Pers Ubiquit Comput.

[31] Metcalf CJ, Edmunds WJ, Lessler J (2015). Six challenges in modeling for public health policy. Epidemics.

[32] Hansen HK, Porter T (2017). What do big data do in global governance?. Glob Gov.

[33] Marathe M, Vullikanti AKS (2013). Computational epidemiology. The challenge of developing and using computer models to understand and control the diffusion of disease through populations. Commun ACM.

[34] Claes F, Kuznetsov D, Liechti R, Von Dobschuetz S, Truong BD, Gleizes A, et al. The EMPRES-i genetic module: a novel tool linking epidemiological outbreak information and genetic characteristics of influenza viruses. Database. 2014; 10.1093/database/bau008. 10.1093/database/bau008 PMC3945526 24608033

[35] Ferrè N, Kuhn W, Rumor M, Marangon SA (2014). Conceptual holding model for veterinary applications. Geospat Health.

[36] Clements ACA, Pfeiffer DU, Otte MJ, Morteo K, Chen LA (2002). Global livestock production and health atlas (GLiPHA) for interactive presentation, integration and analysis of livestock data. Prev Vet Med..

[37] Gelman A, Price NP (1999). All maps of parameter estimates are misleading. Stat Med.

[38] Huck JJ, Whyatt JD, Coulton P (2015). Visualising patterns in spatially-ambiguous point data. JOSIS.

[39] Heller J (2015). Epidemiological and statistical considerations for interpreting and communicating oncology clinical trials. Vet J.

[40] Cook CE, Bergman MT, Finn RD, Cochrane G, Birney E, Apweiler R (2016). The European bioinformatics institute in 2016: data growth and integration. Nucleic Acids Res.

[41] Zhulin IB (2015). Databases and microbiologists. J Bacteriol.

[42] Manzoni C, Kia DA, Vandrovcova J, Hardy J, Wood NW, Lewis PA, Ferrari R. Genome, transcriptome and proteome: the rise of omics data and their integration in biomedical research. Brief Bioinform. 2016:1–17. 10.1093/bib/bbw114. 10.1093/bib/bbw114 PMC6018996 27881428

[43] Jelineck HF, Yatsko A, Stranieri A, Venkatraman S, Bagirov A (2015). Diagnostic with incomplete nominal/discrete data. Airman.

[44] Atzori L, Iera A, Morabito G (2010). The internet of things: a survey. Comput Netw.

[45] Ruiz-Garcia L, Lunadei L, Barreiro P, Robla JIA (2009). Review of wireless sensor technologies and applications in agriculture and food industry: state of the art and current trends. Sensors.

[46] Halachmi I, Guarino M (2016). Editorial: precision livestock farming: a “per animal” approach using advanced monitoring technologies. Animal.

[47] Terrasson G, Llaria A, Marra A, Voaden S (2016). Accelerometer based solution for precision livestock farming: geolocation enhancement and animal activity identification. IOP Conf Ser Mater Sci Eng.

[48] Junkan A, Masip-Bruin X, Amla N. Smart Computing and Sensing Technologies for Animal Welfare: A Syst Rev. arXiv. 2016;1609.00627.

[49] Borgo S, Masolo C. Ontological foundations of DOLCE. In: Poli R, Healy M, Kameas A, editors. Theory and applications of ontology: computer applications. Dordrecht: Springer; 2010. p. 279–95. doi:10.1007/978-90-481-8847-5_13.

[50] Borgo S, Pozza G (2012). Knowledge objects: a formal construct for material, information and role dependences. KMRP.

[51] Pozza G, Borgo S, Oltramari A, Contalbrigo L, Marangon S (2016). Information and organization in public health institutes: an ontology-based modeling of the entities in the reception-analysis-report phases. J Biomed Semant.

[52] Robinson TP, Wint GRW, Conchedda G, Van Boeckel TP, Ercoli V, Palamara E (2014). Mapping the global distribution of livestock. PLoS One.

[53] Greiner M, Gardner IA (2000). Application of diagnostic tests in veterinary epidemiologic studies. Prev Vet Med..

[54] White RW, Tatonetti NP, Shah NH, Altman RB, Horvitz E (2013). Web-scale pharmacovigilance: listening to signals from the crowd. J Am Med Inform Assoc.

[55] Altman RB (2015). Web-scale pharmacovigilance in the discovery of pharmacological effects and toxicity.

[56] Vayena E, Salathé M, Madoff LC, Brownstein JS (2015). Ethical challenges of big data in public health. PLoS Comput Biol.

[57] Pfeiffer DU, Stevens KB (2015). Spatial and temporal epidemiological analysis in the big data era. Prev Vet Med..

[58] Hill AA, Crotta M, Wall B, Good L, O’Brien SJ, Guitian J (2017). Towards an integrated food safety surveillance system: a simulation study to explore the potential of combining genomic and epidemiological metadata. R Soc open sci.

[59] Sivarajah U, Kamal MM, Irani Z, Weerakkody V (2017). Critical analysis of big data challenges and analytical methods. J Bus Res.

[60] Liechti R, Gleizes A, Kuznetsov D, Bougueleret L, Le Mercier P, Bairoch A, Xenarios I. OpenFluDB, a database for human and animal influenza virus. Database 2010. Doi:10-1093/database/baq004. 10.1093/database/baq004 PMC2911839 20624713

[61] Quintarelli E, Rabosio E, Tanca LA (2015). Principled approach to context schema evolution in a data management perspective. Inform. Syst.

[62] Zapater JJS, Escriva DML, Garcià FRS, Durà JJM (2015). Semantic web service discovery system for road traffic information services. Exp Syst Appl.

[63] Crauwels APP, de Konig R, Nielen M, Elbers ARW, Dijkhuinzen AA, Tielen MJMA (2001). Concept for a decision support system based on practical experiences from a national disease emergency: the Dutch experience. Acta Vet Scand.

[64] Nielsen AC (2011). Data warehouse for assessing animal health, welfare, risk management and communication. Acta Vet Scand.

[65] Xu Z, Lee J, Park D, Chung Y (2017). Multidimensional analysis model for highly pathogenic avian influenza using data cube and data mining techniques. Biosyst Eng.

[66] Koç H, Henning E, Jastram S, Starke C (2014). State of the art in context modeling – a systematic literature review. LNBIP.

[67] Mnatsakanyan ZR, Burkom HS, Hashemian MR, Coletta MA (2012). Distributed information fusion models for regional public health surveillance. Inf Fusion.

[68] Dourish P (2004). What we talk about when we talk about context. Personal Ubiquitous Comput.

[69] Wang H-H, Boukiamp F, Elghamrawy T (2011). Ontology-based approach to context representation and reasoning for managing context-sensitive construction information. J Comput Civ Eng.

[70] Grill T, Tscheligi M (2011). Towards a multi-perspectival approach of describing context. LNCS.

[71] Mizoghuci R, Kitamura Y, Borgo S (2012). Towards a unified definition of function. Fr Art Int.

[72] Mizoguchi R, Kitamura Y, Borgo SA (2016). Unifying definition for artifact and biological functions. Appl Ontol.

[73] Chen Z-Y, Fan Z-P, Sun M (2015). Behavior-aware user response modeling in social media: learning from diverse heterogeneous data. Eur J Oper Res.

[74] De Andrés J, Pariente B, Gonzalez-Rodriguez M, Lanvin DF (2015). Towards an automatic user profiling system for online information sites: identifying demographic determining factors. Online Inform Rev.

[75] Haesen R, Snoeck M, Lemahieu W, Poelmans S (2008). On the definition of service granularity and its architectural impact. LNCS.

[76] Tempelman-Kluit N, Pearce A (2014). Invoking the user from data to design. Coll Res Libr.

[77] Pandey S, Srivastava S (2014). Data driven enterprise UX: a case study of enterprise management systems. LNCS.

[78] Kumar RL, Stylianou ACA (2014). Process model for analyzing and managing flexibility in information systems. Eur. J Inf Syst.

[79] Jablonski S, Ruf T, Wedekind H (1990). Optimization of distributed processing by using a flexible data distribution mechanism. In: Rishe N, Navathe S, Tal D, editors. Proceedings PARBASE-90. International Conference on Databases, parallel architectures and their applications.

[80] Allan PD. Decision granularity: preserving uncertainty information in data consolidations. SPE Annual Technical Conference and Exhibition Society of Petroleum Engineers. 2004; 10.2118/90147-MS.

[81] Birkhead GS, Klompas M, Shah NR (2015). Uses of electronic health records for public health surveillance to advance public health. Annu Rev Public Health.

